# Increased TRPV1 Channels and FosB Protein Expression Are Associated with Chronic Epileptic Seizures and Anxiogenic-like Behaviors in a Preclinical Model of Temporal Lobe Epilepsy

**DOI:** 10.3390/biomedicines10020416

**Published:** 2022-02-10

**Authors:** Willian Lazarini-Lopes, Gleice Kelli Silva-Cardoso, Christie Ramos Andrade Leite-Panissi, Norberto Garcia-Cairasco

**Affiliations:** 1 Neuroscience and Behavioral Sciences Department, Ribeirão Preto School of Medicine, University of São Paulo, Ribeirão Preto 14049-900, Brazil; willian.lopes@usp.br; 2 Psychology Department, Faculty of Philosophy, Science, and Letters, University of São Paulo, Ribeirão Preto 14040-901, Brazil; cardoso.gkrs@usp.br (G.K.S.-C.); christie@usp.br (C.R.A.L.-P.); 3 Physiology Department, Ribeirão Preto School of Medicine and Neuroscience and Behavioral Sciences Department, University of São Paulo, Ribeirão Preto 14049-900, Brazil

**Keywords:** epilepsy, anxiety, TRPV1 channels, neuronal activity, neuropsychiatric comorbidity, immunofluorescence, temporal lobe epilepsy, audiogenic kindling

## Abstract

Epilepsies are neurological disorders characterized by chronic seizures and their related neuropsychiatric comorbidities, such as anxiety. The Transient Receptor Potential Vanilloid type-1 (TRPV1) channel has been implicated in the modulation of seizures and anxiety-like behaviors in preclinical models. Here, we investigated the impact of chronic epileptic seizures in anxiety-like behavior and TRPV1 channels expression in a genetic model of epilepsy, the Wistar Audiogenic Rat (WAR) strain. WARs were submitted to audiogenic kindling (AK), a preclinical model of temporal lobe epilepsy (TLE) and behavioral tests were performed in the open-field (OF), and light-dark box (LDB) tests 24 h after AK. WARs displayed increased anxiety-like behavior and TRPV1R expression in the hippocampal CA1 area and basolateral amygdala nucleus (BLA) when compared to control Wistar rats. Chronic seizures increased anxiety-like behaviors and TRPV1 and FosB expression in limbic and brainstem structures involved with epilepsy and anxiety comorbidity, such as the hippocampus, superior colliculus, and periaqueductal gray matter. Therefore, these results highlight previously unrecognized alterations in TRPV1 expression in brain structures involved with TLE and anxiogenic-like behaviors in a genetic model of epilepsy, the WAR strain, supporting an important role of TRPV1 in the modulation of neurological disorders and associated neuropsychiatric comorbidities.

## 1. Introduction

Epilepsies are neurological disorders characterized by chronic epileptic seizures and their consequent neuropsychiatric comorbidities [1,2]. Among them, we can highlight anxiety, which is one of the most common comorbidities associated with epilepsies, not only because of the epileptogenic processes but also as a cause of its exacerbation, which indicates a bidirectional relationship between epilepsies and anxiety [3,4,5]. Therefore, anxiety symptoms, or anxiogenic-like behaviors, may be present either as intrinsic aspects of epileptic seizures or as events related to inter-ictal alterations [6,7,8]. Additionally, anxiety management can overlap epilepsy, and some antiseizure drugs may modulate anxiety in patients with epilepsies, while classical anxiolytic drugs also display antiseizure effects [2,3,9].

The phenomenon of the bidirectionality between neurological disorders and neuropsychiatric comorbidities has been frequently discussed in the literature [10,11]. Studies demonstrated that seizure severity is highly correlated with anxiety and other neuropsychiatric comorbidities, such as depression, in patients with epilepsies, supporting a direct relationship between seizures and affective disorders. Consequently, the diagnosis of epilepsies increases the chances of suicide and further diagnosis of anxiety, but the opposite is also true once patients with anxiety and depressive disorders have a higher risk of developing epilepsies in comparison to those who do not suffer from mood or anxiety disorders [3,5,6,12,13]. Additionally, it is worth noticing that brainstem and limbic structures, such as the periaqueductal gray matter (PAG), superior colliculus, basolateral amygdala nucleus (BLA), and hippocampus, are intrinsically involved with epileptic seizures manifestations [14,15,16,17], as well as with anxiogenic, emotional and defensive behaviors [18,19,20,21], indicating that neuroplastic and functional alterations in these neuronal networks can impact epilepsy and anxiety comorbidity.

Psychiatric and neurological disorders are commonly associated with changes in neural calcium ion signaling pathways. The transient receptor potential vanilloid (TRPV) channel family is composed of seven different subfamilies, and most of their members are closely related to the modulation of calcium influx. The TRPV type-1 (TRPV1) is a non-selective calcium-permeable cation channel with high Ca^2+^ permeability that has been associated with a wide range of biological functions, such as synaptic plasticity, anxiety, fear, stress, thermoregulation, and pain [22,23,24,25,26]. TRPV1 channels are widely expressed in the brain, but cortical and limbic structures present the highest levels of TRPV1 protein and mRNA [27,28]. Moreover, TRPV1 signaling is associated with other neurotransmitter systems related with neuropsychiatric disorders, especially the endocannabinoid [29,30,31], which makes its neurobiological function even more complex to understand.

Since TRPV1 receptors are involved with calcium mobilization, these channels have been shown to be intrinsically involved with synaptic excitability and, consequently, with epileptic seizure susceptibility and manifestation [32,33,34]. Increased mRNA and protein levels of TRPV1 channels were observed in the hippocampus and cortex of patients with temporal lobe epilepsy (TLE) [35] and preclinical models suggest an important role for TRPV1 in seizure susceptibility, especially because its pharmacological activation facilitates seizure manifestation [33,36,37]. Additionally, evidence supports the role of TRPV1 channels located in the brainstem and limbic structures in the modulation of emotional and anxiety-like behaviors [22,38,39].

The Wistar Audiogenic Rat (WAR) strain is a genetic model of epilepsy with animals susceptible to audiogenic seizures (AGS)—for a comprehensive review see [40]. Acute AGS mimics generalized tonic-clonic seizures associated with brainstem hyperactivity, where the inferior colliculus (IC), deep layers of the superior colliculus (DLSC), and dorsal PAG (dPAG) play an important role in seizure susceptibility and manifestation [15,41]. However, during the chronic protocol of AGS, the audiogenic kindling (AK), firstly demonstrated by Marescaux et al. [42], limbic seizures, modulated by cortical and limbic brains sites, coexist with brainstem seizures. Thus, AK in genetically susceptible rodents is considered a model of TLE with limbic recruitment characterized by electrographic and behavioral seizures [43,44,45,46,47,48], similar to those described by Racine in the amygdala kindling protocol [49]. It is worth noting that genetic selection for seizure susceptibility in the WAR strain also selected physiological and behavioral alterations associated with epilepsy related comorbidities [40,50]. Additionally, chronic seizures in WARs induce spatial memory deficits [51] and increase the expression of glutamate receptor subunits [52] and CB1 receptors [53] in limbic structures. Therefore, genetic susceptibility and chronic epileptic seizures are both involved with manifestations of neuropsychiatric comorbidities underlying epilepsy in the WAR strain.

However, the impact of both genetic background and chronic epileptic seizures on anxiety-like behaviors still needs to be appropriately investigated in WARs. Similarly, despite TRPV1 channels being closely related with epilepsies and their comorbidities, possible neuroplastic alterations in their expression have never been analyzed, either in WARs or in other genetic models of audiogenic seizures. Therefore, the present study aimed to investigate endogenous alterations in TRPV1 channels expression in the brainstem and limbic structures involved with seizure susceptibility and anxiogenic-like behaviors in the WAR strain. We also characterized the impact of AK, a model of TLE, on anxiety-like behaviors and TRPV1 and FosB expression in the brainstem and limbic structures involved with the comorbidity epilepsy and anxiety in animals of the WAR strain.

## 2. Materials and Methods

### 2.1. Animals

Adult male Wistar (*n* = 16) and WAR (*n* = 16) rats (2–4 months old) were provided by the Central Vivarium of the University of São Paulo, Ribeirão Preto, and by the Special Rat Strains’ Vivarium of the Ribeirão Preto School of Medicine, respectively. Animals were kept at the Animal Housing Facility of the Physiology Department of the Ribeirão Preto School of Medicine, University of São Paulo, housed in groups of 3–4 animals per cage in a controlled temperature (23 ± 2 °C) under light/dark cycle of 12/12 h. (lights on at 6:00 a.m.), with access to food and water ad libitum. The experimental protocol was approved by the Ethics Committee in Animal Research of the Ribeirão Preto School of Medicine, University of São Paulo (Protocol number: 057/2017; approval date: 2 August 2017).

### 2.2. Chronic Audiogenic Seizure (AGS) Protocol: Audiogenic Kindling (AK)

Chronic AGS were induced as previously described [43,53]. Briefly, animals were placed into an acrylic cylindrical chamber located at a soundproof wood chamber. A small speaker connected to a computer was placed on the top of the acrylic chamber. In every test session, animals were placed into the chamber and after 1 min the sound (110–120 dB; 5–20 kHz) was manually triggered by the researcher and applied until the onset of a tonic seizure, or for a maximum of 60 s if no seizure was observed. Animal behavior was recorded for 1 min before sound, maximum of 1 min during sound exposure, and 1 min after sound. During the AK, WARs (*n* = 8) were submitted to 20 acoustic stimulations for 10 days (2 per day), every morning (8:00–9:00 a.m.) and afternoon (5:00–6:00 p.m.). The apparatus was cleaned with 5% ethanol solution after each test session.

Wistar rats (*n* = 8) were submitted to the same protocol, for two reasons: as a control of the specificity of the AK protocol to induce seizures only in genetically susceptible rats and to investigate if changes induced by the AK in WARs were, in fact, due to the epileptogenic process and not merely due to the chronic sound exposure. Additionally, to investigate possible endogenous alterations in anxiety-like behaviors, chronic neuronal hyperactivity, and TRPV1 expression in the WAR strain, control (non-stimulated) WARs (*n* = 8) and Wistars (*n* = 8) were submitted to a false AK (Sham) protocol, exactly as described for chronic stimulated animals, but the sound was never applied in these groups.

Brainstem seizure severity was analyzed according to the Brainstem index [54], where: 0 = no seizure; 1 = one running; 2 = one wild running (running with jumps and atonic falls); 3 = two wild runnings; 4 = tonic convulsion; 5 = tonic seizure followed by generalized clonic convulsion; 6 = index 5 plus head ventral flexion; 7 = index 6 plus forelimb hyperextension; 8 = index 7 plus hindlimb hyperextension. Limbic seizures were measured according to the Racine scale [49], where: 0 = no seizure; 1 = facial and years myoclonus; 2 = head myoclonus; 3 = forelimb myoclonus; 4 = forelimb myoclonus, followed by elevation; 5 = forelimb myoclonus, followed by elevation and fall.

### 2.3. Behavioral Tests for Anxiety

**Figure 1:**
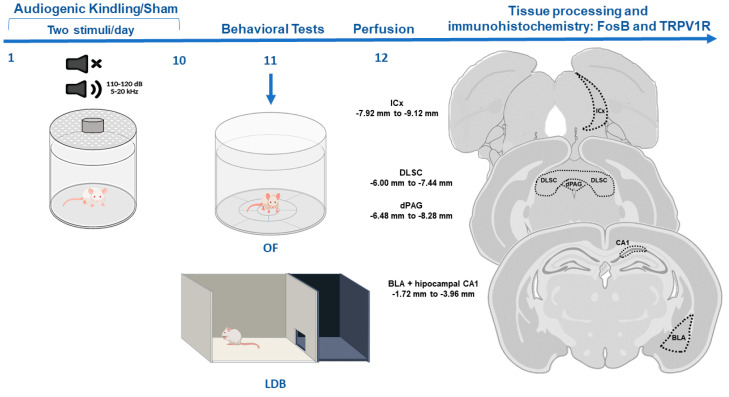
Experimental design. Animals were submitted to the audiogenic kindling (AK) protocol with 20 acoustic simulations. After that, animals were submitted to behavioral tests, and tissue was collected for immunohistochemistry. Abbreviations: OF—open field test, LDB—light/dark box test, ICx—inferior colliculus cortical area, dPAG—dorsal periaqueductal gray matter, DLSC—deep layers of superior colliculus, BLA—basolateral amygdala nucleus, CA1—dorsal hippocampus sub-region.

In the morning (8:00–11:00 a.m.) after the last AGS or sham exposure to the cylinder, animals were submitted to the open-field (OF) test followed by the light/dark box (LDB) test to investigate possible alterations in anxiety-like behaviors (see Figure 1).

The OF consists of an acrylic circular arena (90 cm diameter) with a 40 cm wall, and a floor divided into 12 equal areas. Animals were placed in the center of the apparatus (50 lux) and behaviors were recorded for 5 min by a camera located above the apparatus. The time spent in the central area of the apparatus was calculated and the locomotory activity was measured according to the number of crossings. Duration and frequency of vertical exploratory behaviors (rearings) and grooming were also measured.

The light/dark box (LDB) test was performed 5 min after the OF in a different room. The apparatus consists of an acrylic box with overall dimensions of 100 × 50 × 40 cm (length, width, height) divided into a light and a dark compartment with a doorway (10 × 10 cm) communicating both sides. The lit side (65 lux) has floor and walls painted white, a transparent roof, and corresponds to 2/3 of the size of the apparatus. The dark compartment (0 lux) has floor, walls, and roof all painted black and corresponds to 1/3 of the size of the apparatus. Animals were individually placed in the center of the lit compartment turned back to the doorway separating both compartments. Behavior was recorded for 5 min by a camera located above the apparatus and the following parameters were measured: number of entries and time spent into the lit compartment, latency to the first transition to the dark compartment, latency to the first return to the lit compartment, and frequency and duration of rearings. The number of risk assessment behaviors, such as stretched attempt postures and pokes, were also measured. After each session, the OF and LDB were cleaned with 5% ethanol.

### 2.4. Tissue Processing and Immunohistochemistry

Animals were anesthetized with sodium thiopental (50 mg/kg; i.p.; Abbott, São Paulo, Brazil) and perfused with phosphate-buffered saline (PBS 0.1M, pH 7.4) followed by paraformaldehyde (PFA 4%, pH 7.4) 24 h after behavioral tests. Brains were post-fixed in PFA for 4 h and then cryoprotected in sucrose solution 30% at 4 °C and, after that, they were frozen in isopentane and dry ice. Using a cryostat (Microm HM-505-E, Microm International, Walldorf, Germany), serial coronal sections (40 μm) from the dorsal hippocampus, BLA, DLSC, dPAG, and the cortical area of the IC (ICx) were cut following the coordinates from Paxinos and Watson [55]. Slices were stored in a cryoprotection solution (50% PBS, 30% ethylene glycol, 20% glycerol) until immunohistochemical experiments. Representative images of each structure are illustrated in Figure 1.

Immunofluorescence for TRPV1 channels was performed in the DLSC, dPAG, BLA, and in the CA1 region of the dorsal hippocampus, as we have previously described [56]. Briefly, after washing in PBS, free-floating sections were incubated overnight in a mouse polyclonal antibody that recognizes the C-terminal of the TRPV1 receptor (Abcam, ab203103, Cambridge, UK) diluted in normal goat serum (1:1000). Tissues were washed in PBS and then incubated for 2 h in an anti-mouse IgG Rhodamine B (AP192R) diluted in normal goat serum (1:1000). The slides were mounted in Vectashield mounting medium (Vector Laboratories, Burlingame, CA, USA) and stored at 4 °C. Immunostaining for FosB+ neurons were performed in the ICx, DLSC, dPAG, and BLA as previously described [57]. Briefly, free-floating sections were washed in PBS and incubated overnight in a rabbit polyclonal primary antibody (1:1000; sc-48, Santa Cruz Biotechnology, Dallas, TX, USA) diluted in a 2% solution of bovine serum albumin solution (BSA, Amresco, Solon, OH, USA). On the next day, slices were washed in PSB and incubated for 2 h in a biotinylated secondary antibody anti-rabbit IgG (1:1.000; BA-1000, lot. Zb0318, Vector) diluted in BSA solution. Immunoreactive sites were visualized using the 3,3′-diaminobenzidine (DAB) peroxidase substrate with nickel (SK-4100, Vector). Slices were mounted on glass slides and coverslipped with Permount (Sigma-Aldrich, Inc., St. Louis, MO, USA).

For FosB and TRPV1R immunohistochemical protocols, negative control sections were incubated as described, but without the primary antibody, and immunoreactivity was absent in these sections.

### 2.5. Image Processing and Analysis

Immunoreactive sites were visualized and photographed in a scanning microscope (Olympus BX61VS). Tissues processed for TRPV1R immunofluorescence and FosB+ neurons were analyzed in 400× magnification and the software ImageJ (National Institute of Mental Health, Bethesda, MD, USA) was used for image processing analysis as we previously described [56,57].

To analyze the TRPV1 immunofluorescence images, we used the Integrated Optical Density (IOD) method. Six slices per animal were analyzed for each structure of interest, with a sample of six animals randomly selected per group. The intensity was analyzed using the software ImageJ (https://imagej.nih.gov/ij/ version 1.8.0; accessed on 20 November 2021) and the mean value of the integrated density (the product of the area and the mean gray value) was calculated using the mean value of the three regions of interest (ROI), randomly selected within each structure of each animal. In smaller areas, such as CA1 and dPAG, we used 3 ROIs with 2.500 μm^2^, while in the BLA, DLSC, and IC, 3 ROI with 10.000 μm^2^ were used. For FosB immunostaining, three slices per animal were analyzed for each region of interest, with a sample of five animals randomly selected per group. To analyze the number of FosB+ neurons, we opted for the manual counting method (blind researcher). For each animal, we used the arithmetic mean of the total number of FosB+ cells detected in 3 ROIs (10.000 mm^2^ each one) randomly selected within each structure.

### 2.6. Statistical Analysis

Data were tested for normality using the Shapiro–Wilk test. Statistical analysis was performed using Two-Way ANOVA (variables: STRAIN and AK) followed by post hoc Tukey’s test. Data were expressed as mean ± standard error of the mean, and the software GraphPad Prism 9.0 (GraphPad Software, Inc., La Jolla, CA, USA) was used to perform statistical analysis and to create the graphics. Significant differences: *p* < 0.05.

## 3. Results

### 3.1. Audiogenic Kindling (AK) Progression

**Figure 2:**
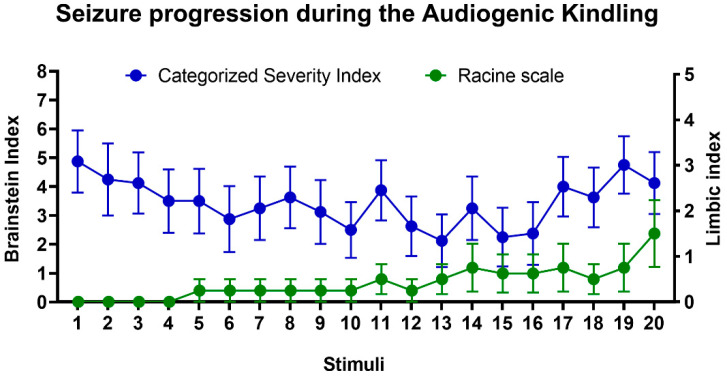
Evolution of audiogenic kindling (AK) in WARs. Mean of brainstem (blue circles) and limbic (green circles) seizure severity during AK. Only brainstem generalized tonic-clonic seizures were detected at the beginning of the protocol; however, during the AK, limbic seizures coexist with those that originated from the brainstem. Data are represented by mean ± standard error mean (*n* = 8/group).

In the beginning of the protocol, WARs developed brainstem AGS in response to intense sound stimulation; seizures were characterized by wild running with jumps and atonic falls followed by generalized tonic-clonic seizure behaviors, such as forelimb hyperextension, partial or generalized clonic seizures. During AK, limbic seizures coexist with those that originated from brainstem structures; this phenomenon is illustrated by the appearance of novel clonic seizure behaviors such as those described by Racine [49], similar to facial and forelimb myoclonus, followed by body elevation and fall (Figure 2). All WARs developed AGS during the AK protocol and 4/8 rats from the WAR-AK group developed severe limbic seizures (Racine ≥ 4) during the AK and were classified as forebrain-recruited; the remaining four WARs from this group developed consistent generalized tonic-clonic seizures, but limbic seizures were not detected. Wistar rats submitted to chronic auditory stimulation did not develop AGS.

### 3.2. Open-Field (OF) Test

**Figure 3:**
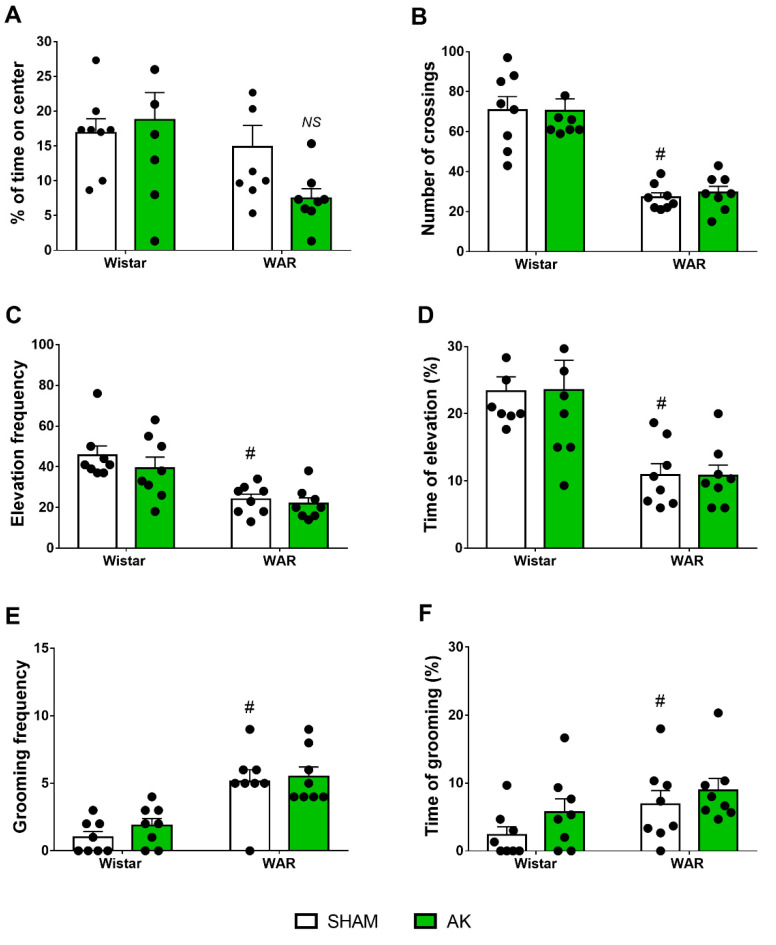
Assessment of anxiety-like behavior in the open-field (OF) test. The measures analyzed were (**A**) percentage of time in the center, (**B**) the number of crossings, (**C**) frequency of elevation, (**D**) time of elevation, (**E**) frequency of grooming, and (**F**) time of grooming. The behavioral tests in the OF were performed during 5 min on the 11th experimental day. ^#^ *p* < 0.05 Tukey test comparing WAR-Sham and Wistar-Sham groups. NS = non-significant difference. Data are expressed as means ± standard error mean. N = 8/group.

Results from the OF test are presented in Figure 3. Firstly, regarding the time in the center of the OF, two-way ANOVA showed a significant effect of strain with WARs spending less time in the center (F1, 28 = 5.626; *p* = 0.0248), but no AK effect (F1, 28 = 0.9779; *p* = 0.3312) was detected, neither in WARs nor in Wistars.

Regarding the locomotor activity during the OF test, two-way ANOVA showed a powerful strain effect, with WAR presenting less crossings than Wistars (F1, 28 = 73.23; *p* < 0.0001), but AK showed no significant effects (F1, 28 = 0.04102; *p* = 0.8410).

Vertical exploratory activity was impaired in WARs in comparison with Wistars in both parameters, frequency (F1, 28 = 23.44; *p* < 0.0001) and percentage of time (F1, 28 = 21.52; *p* < 0.0001). However, neither AK (WARs) nor the chronic exposure to intense sound stimulation (Wistars) had any effect on frequency (F1, 28 = 1.113; *p* = 0.3004) or percentage of time (F1, 28 = 8.439 × 10^−21^; *p* > 0.9999) of the vertical exploratory behaviors in WARs and Wistar, respectively.

Two-Way ANOVA showed a significant strain effect regarding grooming behavior, with WARs expressing increased number (F1, 28 = 35.13; *p* < 0.0001) and duration (F1, 28 = 4.756; *p* = 0.0377) of grooming patterns in comparison to Wistars. However, as for the previous parameters, neither AK (WARs) nor the chronic exposure to intense sound stimulation (Wistar) changed the frequency (F1, 28 = 0.9138; *p* = 0.3473) or the percentage of time of grooming behaviors (F1, 28 = 2.324; *p* = 0.1386).

### 3.3. Light Dark Box (LDB) Test

**Figure 4:**
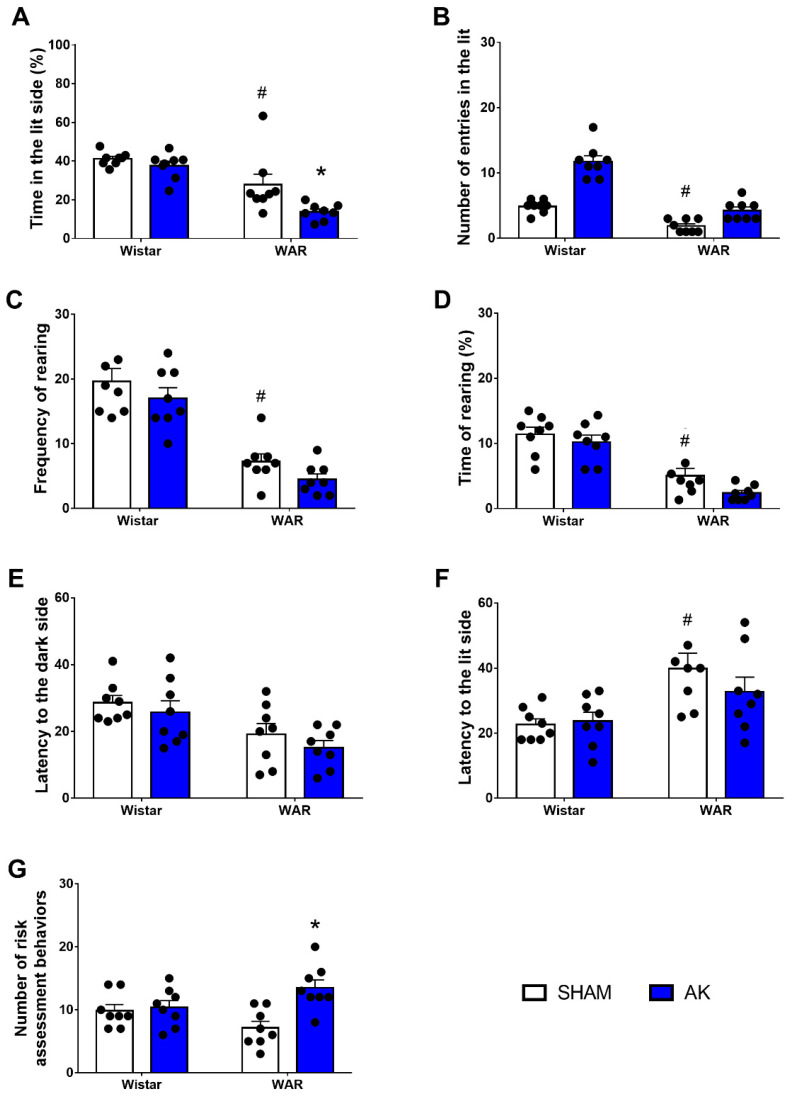
Assessment of anxiety-like behavior in the light/dark box (LDB) test. The measures analyzed were (**A**) percentage of time in the lit side of the box, (**B**) the number of crossings, (**C**) frequency rearing, (**D**) time of rearing, (**E**) latency to the dark side, (**F**) latency to the lit side, and (**G**) risk assessment behaviors. The LBD test was performed during 5 min on the 11th experimental day. * *p* < 0.05 Tukey test comparing WAR-AK and WAR-Sham. ^#^ *p* < 0.05 Tukey test compared to WAR-Sham and Wistar-Sham. Data are expressed as mean ± standard error mean. N = 8/group.

Results from the LDB test are illustrated in Figure 4. Firstly, regarding the percentage of time spent in the lit side of the LDB, Two-Way ANOVA revealed significant effects of both, strain (F1, 28 = 35.9; *p* < 0.0001) and AK (F1, 28 = 7.895; *p* = 0.0089). Tukey’s post-test revealed that WARs spent less time spent in the lit side than Wistars (*p* = 0.0262). Additionally, the time in the lit side was reduced even more in WARs after AK (*p* = 0.0184), but the AK protocol did not change the time spent in the lit side of the LDB in Wistar rats (*p* = 0.8502).

Two-Way ANOVA showed a significant strain effect in the number of entries in the lit side of the LDB, with WARs presenting a reduced number of entries in the lit side in comparison to Wistars (F1, 28 = 70.10; *p* < 0.0001). Again, the AK protocol showed no effects on the number of entries in the lit side, neither in WARs nor in Wistars (F1, 28 = 0.9333; *p* = 0.3423).

Similar to that observed in the OF test, vertical exploratory activity was impaired in WARs when compared to Wistars in both, frequency (F1, 28 = 70.23; *p* < 0.0001) and duration (F1, 28 = 54.96; *p* < 0.0001). However, AK showed no significant impact, either in frequency (F1, 28 = 3.279; *p* = 0.0809) or in duration (F1, 28 = 4.088; *p* = 0.528) of vertical exploratory behaviors.

Regarding the first latency to the dark compartment, two-Way ANOVA showed a significant strain effect with WARs presenting a shorter latency than Wistars (F1, 28 = 12.86; *p* = 0.0013). The AK protocol did not modify the latency to the dark compartment (F1, 28 = 1.501; *p* = 0.2317). The latency to the first return to the lit compartment was significantly increased in WARs in comparison to Wistars (F1, 28 = 13.75; *p* = 0.0009), Multiple comparisons test showed that animals from the WAR-Sham group had a higher latency to their first return to the lit compartment in comparison to Wistar-Sham (*p* = 0.1).

Risk assessment behaviors were measured based on the number of stretched attempt postures and pokes displayed by animals. Despite no significant strain effect (F1, 28 = 0.02965; *p* = 0.8645), there is an effect of the AK protocol (F1, 28 = 9.965; *p* = 0.0038), as well as an interaction between both factors (F1, 28 = 7.277; *p* = 0.0117). Multiple comparisons analysis showed an increased number of risk assessment behaviors in WARs submitted to AK in comparison to the WAR-Sham group (*p* = 0.0016).

### 3.4. Immunohistochemistry for FosB+ Neurons

**Figure 5:**
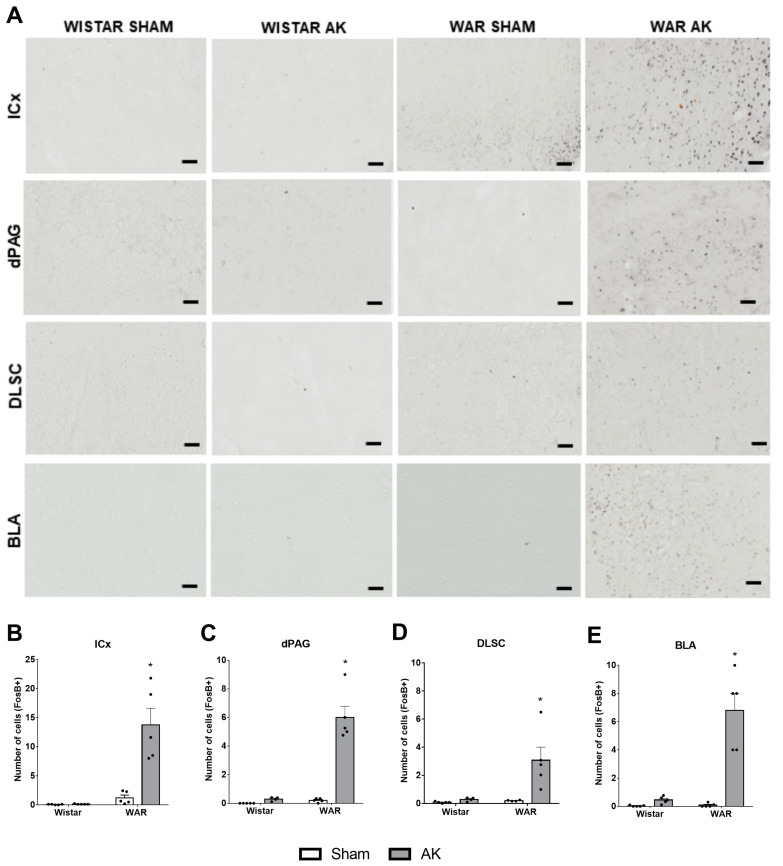
FosB immunostaining after the audiogenic kindling (AK) protocol. (**A**) Representative images of every analyzed area in each experimental group. FosB+ neurons are characterized by black dots, which indicate the nucleus of each immunopositive neuron. (**B**) inferior colliculus cortical area—ICx. (**C**) dorsal periaqueductal gray matter—dPAG. (**D**) deep layers of superior colliculus—DLSC. (**E**) basolateral amygdala nucleus—BLA. * *p* < 0.05 Tukey test compared to WAR-Sham. Data are expressed as mean ± standard error mean. N = 4–5/group. Scale bars = 200 µm.

Results from FosB immunostaining are illustrated in Figure 5. In the ICx, two-way ANOVA showed strain (F1, 16 = 26.93; *p* < 0.0001) and AK effects (F1, 16 = 19.67; *p* = 0.0004), besides an interaction between both factors (F1, 16 = 19.21; *p* = 0.0005). Post hoc analysis revealed that the WAR-AK group presented an increased number of FosB+ neurons in comparison to all the other experimental groups (*p* < 0.0001).

In the DLSC significant strain (F1, 14 = 7.539; *p* = 0.0158) and AK effects (F1, 14 = 8.540; *p* = 0.0111) were detected, as well as a significant interaction between both factors (F1, 14 = 6.360; *p* = 0.0244). Additionally, post hoc analysis showed that the WAR-AK group presented an increased number of FosB+ neurons in the DLSC when compared to all the other groups (*p* ≤ 0.0108).

FosB expression in the dPAG was significantly affected by strain (F1, 15 = 50.33; *p* < 0.0001) and AK effects (F1, 15 = 53.37; *p* < 0.0001). Two-way ANOVA also detected an interaction between both factors (F1, 15 = 43.54; *p* < 0.0001). Post hoc analysis showed significant differences only when comparing the WAR-AK group with the other experimental groups (*p* < 0.0001).

Finally, regarding the FosB expression in the BLA, significant strain (F1, 16 = 28.09; *p* < 0.0001) and AK effects (F1, 16 = 34.97; *p* < 0.0001) were detected. Additionally, two-way ANOVA showed significant interaction between strain and AK (F1, 16 = 27.17; *p* < 0.0001). As observed in the other structures, post hoc analysis showed that the WAR-AK group presented an increased number of FosB+ neurons in comparison to all the other groups (*p* < 0.0001).

### 3.5. Immunofluorescence for TRPV1 Channels

**Figure 6:**
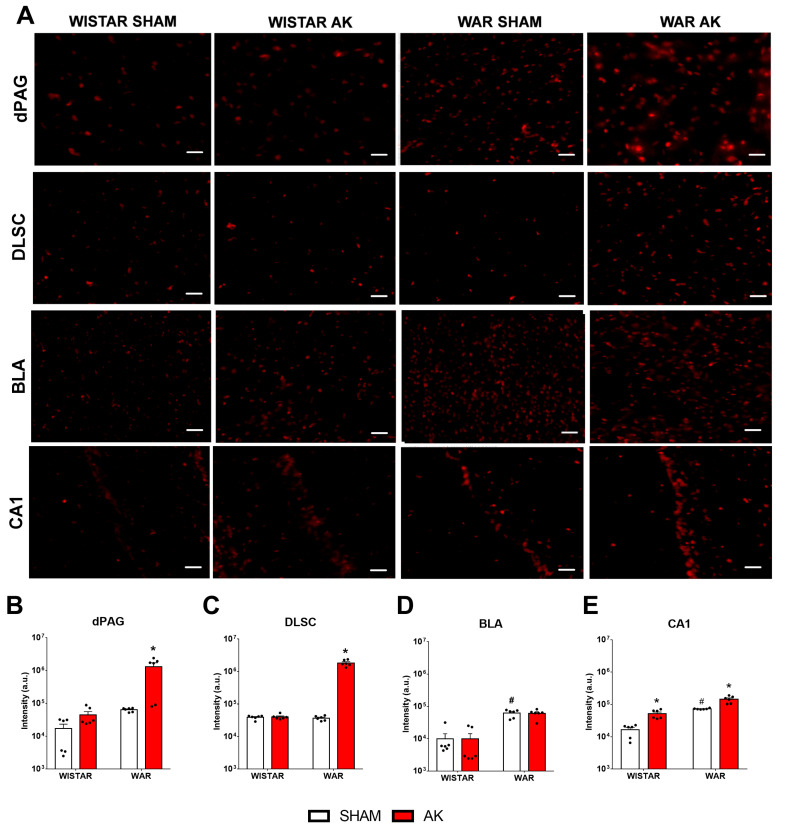
Immunofluorescence for TRPV1 channels after the audiogenic kindling (AK) protocol. (**A**) Representative images of every analyzed area in each experimental group. (**B**) dorsal periaqueductal gray matter—dPAG. (**C**) deep layers of superior colliculus—DLSC. (**D**) basolateral amygdala nucleus—BLA. (**E**) CA1 area of the dorsal hippocampus. ^#^ *p* < 0.05 Tukey test compared to Wistar-Sham. * *p* < 0.05 Tukey test compared to WAR-Sham. N = 6 animals/group. Scale bars = 200 µm.

Results from immunofluorescence for TRPV1 channels are presented in Figure 6. Two-way ANOVA revealed significant strain (F 1, 20 = 70.05; *p* < 0.0001) and AK (F1, 20 = 70.17; *p* < 0.0001) effects of TRPV1R immunofluorescence in the DLSC. Additionally, a significant interaction between strain and AK (F1, 20 = 70.40; *p* < 0.0001) was detected. Post hoc analysis showed no difference between WAR-Sham and Wistar-Sham groups (*p* > 0.9999), however, chronic seizures increased TRPV1 expression in the DLSC in the WAR-AK group in comparison to all experimental groups (*p* < 0.0001). Chronic exposure to intense sound stimulation did not change TRPV1 expression in the DLSC of Wistar rats (*p* > 0.9999).

In the dPAG, significant strain (F1, 20 = 10.71; *p* = 0.0038) and AK (F1, 20 = 10.07; *p* = 0.0048) effects were observed in TRPV1R expression. Significant interaction between both factors was detected (F1, 20 = 9.21; *p* = 0.0065). Post hoc analysis showed no difference between Wistar and WAR Sham groups (*p* = 0.9983), but chronic seizures increased TRPV1R expression in the WAR-AK group in comparison to all experimental groups (*p* < 0.0015). Chronic exposure to intense sound stimulation did not affect TRPV1R expression in Wistars (*p* = 0.9997).

In the BLA, significant strain (F1, 20 = 79.01; *p* < 0.0001), but no AK (F1, 20 = 0.0071; *p* = 0.9337) effects were detected. No significant interaction was detected by two-way ANOVA (F1, 20 = 0.0064; *p* = 0.9370). Post hoc analysis revealed that WARs present increased TRPV1R expression than Wistars, regardless of the experimental condition (*p* < 0.0001). However, neither the chronic seizures in WARs (*p* = 0.9994), nor the chronic exposure to intense sound stimulation in Wistars (*p* > 0.9999) changed TRPV1R expression in the BLA.

In the CA1 of the dorsal hippocampus, two-way ANOVA revealed significant strain (F1, 20 = 87.47; *p* < 0.0001) and AK (F1, 20 = 44.74; *p* < 0.0001) effects. Significant interaction between factors were detected (F1, 20 = 4,862; *p* = 0.0393). Post hoc analysis demonstrated endogenous differences between non-stimulated Wistar and WARs, with WARs presenting increased TRPV1R expression (*p* = 0.0003). Additionally, AK increased TRPV1R expression in CA1 of WARs (*p* < 0.0001), as well as the chronic exposure to intensity sound stimulation increased TRPV1R immunofluorescence in CA1 of Wistars (*p* = 0.0229).

## 4. Discussion

In the present study, we demonstrated that increased TRPV1 immunoreactivity in limbic brain structures, such as the hippocampus and BLA, is associated with genetic susceptibility to epileptic seizures and increased anxiety-like behavior in a genetic model of epilepsy, the WAR strain. Additionally, the AK protocol, a preclinical model of TLE, increased anxiety-like behaviors and TRPV1 immunoreactivity in the dPAG, DLSC, and CA1 in the WAR-AK group compared to control animals. Supporting these alterations, WARs submitted to chronic seizures also displayed an increased number of FosB+ neurons in brainstem and limbic regions related to seizure and anxiety-like behavior manifestation, such as the ICx, dPAG, DLSC, and BLA, indicating increased chronic hyperactivity in these areas.

AK progression in WARs developed similarly as previously described [43,44,58]. Initially, acoustic stimulation induced generalized tonic-clonic seizures, but during the AK, a preclinical model of TLE [43], epileptogenic events modify seizures patterns, which leads to the expression of limbic seizures typical of forebrain recruitment [44,46,58]. It is worth noting that Wistars (non-epileptic control rats) submitted to the AK protocol did not develop AGS, and the chronic high intense sound stimulation had no impact on anxiety or chronic neuronal hyperactivity and only a minor effect on TRPV1 expression. The increased TRPV1 expression in the CA1 of Wistars chronically stimulated was, initially, an unexpected result, especially because it was not associated with any FosB or anxiogenic-like alterations. However, previous data showed that hippocampal TRPV1 signaling is an important mechanism of stress adaptation, once stress effects on hippocampal synaptic activity and spatial memory were prevented by focal and systemic capsaicin administration [59]. Therefore, the increased TRPV1 expression in the CA1 of Wistar rats submitted to chronic intense sound stimulation could be a reflex of adaptive neuroplastic changes in response to chronic stressful situations, such as intense sound exposure.

To our knowledge, the present study is the first to investigate neuroplastic alterations in TRPV1 channels associated with seizure susceptibility in a genetic model of epilepsy, but similar neuroplastic changes have already been reported in other preclinical models. TRPV1 knock-out mice were less susceptible to febrile seizures than control mice; however, seizure development increased TRPV1 mRNA and protein levels in the cortex and hippocampal formation of control mice [60]. Increased TRPV1 protein levels were detected in the dentate gyrus of mice during the acute and chronic phase of the pilocarpine-induced *Status Epilepticus* (SE) model [34,36]. Additionally, bath application of capsaicin increased synaptic transmission in hippocampal slices from epileptic rats but showed no effect on basal synaptic transmission in tissue from control animals [34]. Thus, our results of increased TRPV1 expression in the BLA and dorsal hippocampus agree with previous studies that used febrile and chemical models of epileptic seizures. Furthermore, we also measured TRPV1 immunofluorescence in brainstem structures involved with generalized tonic-clonic seizures and, similar as observed in the BLA, increased TRPV1 expression was detected in the dPAG and DLSC. Therefore, our results suggest an important role of TRPV1 channels in brainstem generalized tonic-clonic and limbic seizures, and these neuroplastic alterations in the BLA, when associated with increased FosB+ neurons, might be related to the intensification of anxiogenic behavior and chronic seizures manifestation.

Previous studies reported anxiogenic-like behaviors in several genetic models of epilepsies. Similar to the results from the present study, animals from the Genetically Epilepsy Prone Rats (GEPR) and Krushinsky-Molodkina (KM), which are also strains genetically susceptible to AGS, display increased anxiety- and depressive-like behaviors [61,62], supporting the presence of emotional behavioral alterations that mimic neuropsychiatric comorbidities in those models. Here, it is crucial to mention that although it is still unclear if alterations in TRPV1 expression and functionality are involved with emotional behavioral alterations found in GEPRs [61], its antagonism with capsazepine have already been shown to attenuate AGS in male and female GEPRs [63]. Furthermore, genetic models of childhood absence epilepsy, such as the Wistar Albino Glaxo from Rijswijk (WAG/Rij) and the Genetic Absence Epilepsy Rats from Strasbourg (GAERS) strains, display increased anxiety- and depressive-like behaviors [64,65]. Similarly, increased anxiogenic-like behaviors and cognitive impairments were detected in the Scn1a +/− mouse model of Dravet syndrome [66], as well as in chemical models of limbic seizures induced by chronic and sub-chronic pentylenetetrazole (PTZ) administration [67,68]. Anxiogenic-like behaviors were also observed in rodents submitted to the pilocarpine-induced SE during the acute (6–10 days after SE) and chronic (3 and 10 months after SE) phase of the model, suggesting that increased anxiogenic-like behavior may underlie epileptogenic events that lead to further seizure manifestation [69,70,71]. Therefore, the present results are in line with previous data and support the presence of the neuroplastic changes in TRPV1 expression underlying the comorbidity of epilepsy and anxiety in genetic and chemical models of epilepsies.

Strikingly, in a previous study, authors performed several behavioral analyses in Wistar and WAR rats, including sucrose preference, forced swimming test, social preference, aversive memory tests, OF, and EPM [72]. WARs displayed several behavioral alterations in these tests, but no difference was detected in the exploration of the open and enclosed arms of the EPM. Briefly, a study conducted with the original WAR strain [50] detected decreased exploration of the open arms in the EPM. However, results from Castro et al. [72] are from a branch of the original WARs, maintained in a different location (Federal University of Minas Gerais, Belo Horizonte, Brazil). Powell et al. [73], compared seizures, behavior, and brain morphology between four different GAERs colonies and they detected variations in seizure severity, anxiety- and depressive-like behaviors. Such behavioral divergences could be associated, therefore, with epigenetic signatures (e.g.,: DNA methylation, histone modifications, and noncoding RNAs). We speculate that several of these factors are also involved in the variations observed between WARs from different colonies.

Neuronal networks involved with generalized tonic-clonic and limbic seizures manifestations are closely related to emotional and anxiogenic-like behaviors [15,18,19]. This relationship can be particularly observed in the WAR strain. Once the IC is the main brainstem structure involved with auditory processing associated with seizures, it plays a key role in AGS susceptibility and manifestation [74], especially the ICx area, which presents increased epileptogenic activity and sends glutamatergic projections to the dPAG and DLSC [75,76,77]. Thus, the hypersynchronism and hyperactivity in these structures mediate the motor manifestation of generalized tonic-clonic seizures [15,78]. During AK, chronic brainstem hyperactivity, especially in excitatory projections from the ICx to the medial geniculate body and then to the BLA, hippocampal formation, and several cortical areas, lead to the phenomenon called limbic recruitment. Once recruited, forebrain regions such as cortical and limbic areas display epileptogenic activities associated with the expression of electrographic and behavioral limbic seizures [42,43,44,45,46,48,58].

Differently from other models of TLE, such as the pilocarpine-induced SE [79], in the AK, the hippocampus does not present neuronal loss, and it is extremely resistant to recruitment; however, c-Fos immunostaining becomes evident in the BLA after 5–10 AGS, when limbic seizures start to coexist with brainstem seizures [76]. Nevertheless, the hippocampus of WARs does present neuroplastic alterations associated with epilepsy susceptibility and neuropsychiatric alterations, such as reduced GABAergic currents in hippocampal neurons [80,81], increased CB1 expression [53], hippocampal hyperplasia [82], and increased calcium concentration in hippocampal synaptosomes [83]. The amygdaloid complex, especially the BLA, is probably the limbic structure most susceptible to AK epileptogenic events, presenting neuronal loss and increased neo-Timm+ staining (Zn^+^ sprouting) [58], as well as increased neuronal hyperactivity and CB1 expression [53,57].

Additionally, fully kindled GEPR-9s have their post-tonic clonic seizure abolished after administration of a selective adenylyl cyclase inhibitor directly into the lateral amygdala nucleus [84]. Similar results were also observed in kindled GEPR-3s who had limbic seizures prevented by administering the NMDA receptor antagonist AP7 into the same structure [85]. Thus, the increased TRPV1 expression endogenously detected in the hippocampus and BLA of WARs is in line with previous neuroplastic alterations and add new information regarding epilepsy susceptibility and calcium mobilization in these limbic brain sites.

Although the dPAG and DLSC are involved with AGS expression [16,17,41,86], they are also intrinsically associated with anxiety-like, defensive, and panic-like behaviors [87,88,89]. Pharmacological manipulations of TRPV1 channels in these structures have also been shown to modulate anxiety. Capsaicin administration into the dPAG increased anxiogenic-like behaviors in rodents [90,91], while TRPV1 antagonism with capsazepine induced the opposite effects [91]. Although TRPV1 channels from neurons located in the superior colliculus have been shown to play an important role in long-term synaptic plasticity [92], there is still a lack of studies investigating their role on either epilepsy or anxiety. Therefore, our results indicate that increased TRPV1 expression and signaling in the dPAG and DLSC of chronically stimulated WARs may be involved with the increased number of FosB+ neurons in the same area. Together these neuroplastic alterations might play an important role on seizure susceptibility and anxiogenic-like behaviors displayed by epileptic rats.

In forebrain sites, activation of TRPV1 located at the dorsal hippocampus induced anxiogenic-like behaviors [29], while its antagonism in the ventral hippocampus induced anxiolytic-like behaviors [39]. Furthermore, anxiolytic-like effects induced microinjection of anandamide within the BLA were shown to be dependent on previous antagonism of TRPV1 located in the same structure, suggesting that anxiolytic effects were associated with CB1 activation [93]. While the local activation of CB1 inhibits calcium channels and excitatory neuronal activity [94], TRPV1 activation promotes calcium influx and glutamate release [95,96]. Thus, once TRPV1R are activated by the endogenous cannabinoid anandamide and its signaling leads to intracellular calcium mobilization and increasing neuronal excitability [31], its increased expression in the analyzed brain structures may explain, at least in part, the genetic susceptibility to epilepsy and the anxiogenic-like behaviors displayed by WARs. Therefore, given the dual role of these receptors in the modulation of emotional behaviors [97], the results from the present study may be associated with the increased CB1 expression previously detected in limbic brain sites of WARs [53], where CB1 receptors could be up-regulated in response to the increased excitability related to increased TRPV1 expression and signaling.

Although we did not perform a co-localization immunohistochemical study to verify if increased TRPV1R immunoreactivity was present in FosB+ neurons, our results from both immunohistochemical protocols, coupled with behavioral analysis, suggest that the increased neuronal hyperexcitability detected in chronically stimulated WARs is due to the increased TRPV1 expression and activity in these neurons, increasing calcium signaling and neuronal firing. This explanation is supported by the increased number of FosB+ neurons in the ICx, DLSC, dPAG, and BLA, and the increased TRPV1R expression in the dPAG, DLSC, BLA, and hippocampal CA1 region. Furthermore, using a genetic model coupled with a chronic protocol of epileptic seizures (AK) and histological analyses are important tools for translational neuroscience, especially in the context of neuropsychiatric comorbidities commonly observed in patients with epilepsies. Intriguingly, several of the mentioned neural substrates or neuronal networks are extensively overlapped when we study either defense systems associated with panic or flight behaviors [19,87,88,98] or those involved in the behavioral and electrographic expression of epileptic seizures [15,16,17,57]. The actual meaning of these overlayed substrates in the context of behavioral and evolutive neuroscience, although not the main goal of the current study, deserves further examination. Indeed, that challenge has been taken brightly in a recent comprehensive literature review by Inga Poletaeva´s research group [99].

In conclusion, increased TRPV1 channels expression in the hippocampus and BLA are associated with seizure susceptibility and anxiogenic-like behaviors in the WAR strain. Additionally, epileptogenic events underlying the limbic recruitment intensified anxiogenic-like behaviors and increased TRPV1 expression in the brainstem and limbic regions involved with the modulation of seizures and emotional behavior. Therefore, the present study added new neuropathological information about the epileptogenic process underlying the limbic recruitment associated with AK, a TLE model, and the consequent development of neuropsychiatric comorbidities. These data shade light on an important role for TRPV1 channels and calcium mobilization in the regulation of endogenous mechanisms related to seizure susceptibility in animals genetically susceptible to epilepsies, supporting a key role for this receptor in epilepsy and anxiety as comorbidities.

## Data Availability

The data presented in this study are available on request from the corresponding author.
